# Styryl Quinazolinones as Potential Inducers of Myeloid Differentiation via Upregulation of C/EBPα

**DOI:** 10.3390/molecules23081938

**Published:** 2018-08-03

**Authors:** Radhakrishnan Sridhar, Hisashi Takei, Riyaz Syed, Ikei S. Kobayashi, Liu Bee Hui, Ahmed Kamal, Daniel G. Tenen, Susumu S. Kobayashi

**Affiliations:** 1 Cancer Science Institute of Singapore, National University of Singapore, Singapore 117599, Singapore; csiradha@nus.edu.sg (R.S.); csilbh@nus.edu.sg (L.B.H.); 2 Department of Medicine, Beth Israel Deaconess Medical Center and Harvard Medical School, Boston, MA 02215, USA; m14702054@gunma-u.ac.jp (H.T.); ikobayas@bidmc.harvard.edu (I.S.K.); 3 Department of Hematology, Gunma University Graduate School of Medicine, Maebashi, Gunma 371-8511, Japan; 4 Medicinal Chemistry and Pharmacology, CSIR-Indian Institute of Chemical Technology, Hyderabad 500 007, India; riyazsd@gmail.com (R.S.); ahmedkamal1915@gmail.com (A.K.); 5 Department of Chemistry, Jawaharlal Nehru Technological University, Kukatpally, Hyderabad-500 085, India; 6 School of Pharmaceutical Education and Research (SPER), Jamia Hamdard, New Delhi-110 062, India; 7 Harvard Stem Cell Institute, Harvard Medical School, Boston, MA 02215, USA; 8 Division of translational Genomics, Exploratory Oncology Research and Clinical Trial Center, National Cancer Center, Kashiwa, Chiba 277-8577, Japan

**Keywords:** myeloid differentiation, CCAAT/enhancer binding protein α

## Abstract

The CCAAT enhancer-binding protein α (C/EBPα) plays an important role in myeloid cell differentiation and in the enhancement of C/EBPα expression/activity, which can lead to granulocytic differentiation in acute myeloid leukemia (AML) cells. We found that styryl quinazolinones induce upregulation of C/EBPα expression, and thereby induce myeloid differentiation in human myeloid leukemia cell lines. We screened a series of active styryl quinazolinones and evaluated the structure–activity relationship (SAR) of these small molecules in inducing C/EBPα expression—thereby prompting the leukemic cells to differentiate. We observed that compound **78** causes differentiation at 3 μM concentration, while **1** induces differentiation at 10 μM concentration. We also observed an increase in the expression of neutrophil differentiation marker CD11b upon treatment with **78**. Both the C/EBPα and C/EBPε levels were found to be upregulated by treatment with **78**. These SAR findings are inspiration to develop further modified styryl quinazolinones, in the path of this novel differentiation therapy, which can contribute to the care of patients with AML.

## 1. Introduction

Knowledge of the pathogenesis of acute myeloid leukemia (AML) has seen great progress due to recent advances in genetic studies. However, the long-term survival of AML patients is still unsatisfactory [1]. A block in differentiation is one of the characteristics of leukemia and is seen in all AML subtypes. Currently, all-trans retinoic acid (ATRA) is the therapy of choice for the induction of differentiation and the apoptosis of t(15;17)-positive acute promyelocytic leukemia (APL) cells [2]. While ATRA treatment induces remission and constitutes a cure in nearly 70% of APL patients, it has no effect on other myeloid leukemias [3]. Therefore, a novel differentiation therapy is sorely needed. The CCAAT enhancer-binding protein α (C/EBPα) is an essential transcription factor for the differentiation of cells in the liver, lung, adipose tissues, and bone marrow [4,5,6,7]. In the hematopoietic system, C/EBPα is expressed in myeloid cells and is required for granulocytic and monocytic differentiation [8]. We have previously reported that C/EBPα expression is necessary and sufficient for neutrophil differentiation [9]. Therefore, when C/EBPα expression or function is perturbed in myeloid progenitor cells, it can block differentiation and eventually lead to myeoid leukemias. We demonstrated that C/EBPα somatic mutations are detected in approximately 7% of AML patients [10,11]. These mutations result in either the premature termination of C/EBPα translation or perturb DNA binding, thus supporting the notion that attenuated C/EBPα activation is associated with AML.

**Figure 1:**
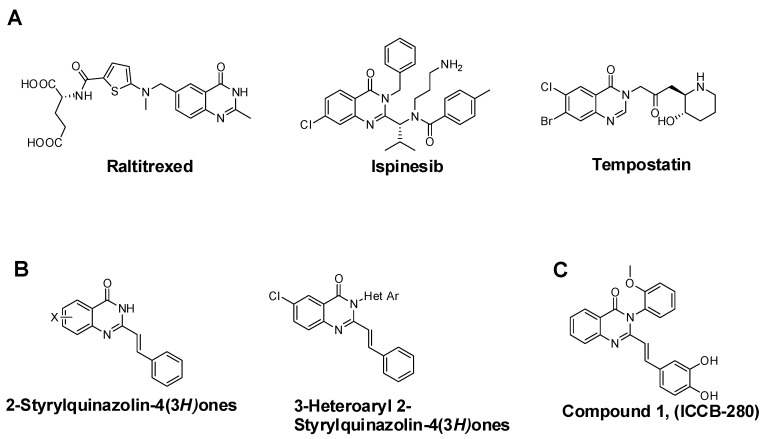
Quinazolinone compounds; (**A**) quinazolinone drugs in the market and in early development [13,14,15], (**B**) Styryl quinazolinones explored as tubulin polymerization inhibitors [18,19,20], (**C**) Styryl quinazolinone **1**, (**ICCB-280**) hit from small molecules C/EBPα inducer screens [22].

The quinazolinone scaffold (Figure 1) is widely represented among bioactive molecules [12]. For example, raltitrexed, a thymidylate synthase inhibitor, has been used for colorectal cancer and malignant mesothelioma [13]. Ispinesib and tempostatin are in development for treatment of various cancers [14,15]. Several styryl quinazolinones specifically target methicillin-resistant staphylococcus aureus infections [16,17]. Hamel et al. reported, in the L1210 leukemia model [18,19,20], 2-phenyl quinazolinones and styryl quinazolinones to be inhibitors of tubulin polymerization and in vivo tumor growth. Another report found styryl quinazolinones to cause the shortening of telomeres [21]. Based on our hypothesis, that increased C/EBPα expression and/or activity in AML cells will stimulate the differentiation of leukemic cells, we screened several libraries of compounds for C/EBPα induction activity using a reporter assay [22]. We found that styryl quinazolinone analogue compound **1** (2-[(*E*)-2-(3,4-dihydroxyphenyl)vinyl]-3-(2-methoxyphenyl)-4(3*H*)-quinazolinone increased C/EBPα activity, which in turn enhanced differentiation leading to growth arrest and the apoptosis of leukemic cells.

In this study, we screened a library of styryl quinazolinones to find more potent inducers of C/EBPα upregulation and differentiation. We also evaluated the structure-activity relationship (SAR) of the styryl quinazolinones in terms of their ability to upregulate C/EBPα and to induce granulocytic differentiation in human myeloid leukemia cells.

## 2. Results and Discussion

**Scheme 1:**
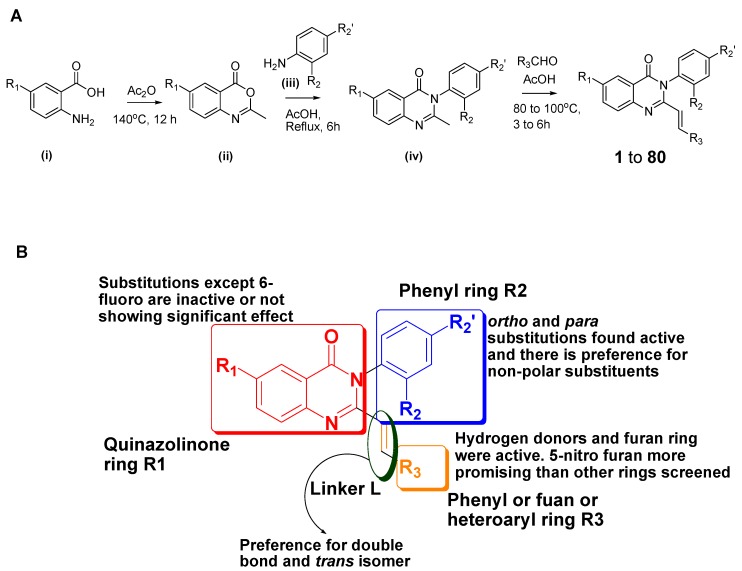
Styryl quinazolinones synthesis, structure, and activity. (**A**) General synthetic protocol for Styryl Quinazolinone synthesis. (**B**) SAR of styryl quinazolinones as granulocytic differentiation inducers.

We have earlier identified styryl quinazolinone **1** as a granulocytic differentiation inducer in myeloid leukemia [21]. In order to arrive at more detailed SAR on this scaffold, we evaluated a library of 80 styryl quinazolinones (Scheme 1A) using various approaches, namely the Wright-Giemsa staining, to assess changes in morphology, nitroblue tetrazolium (NBT) reduction assay to evaluate granulocyte function, and trypan-blue staining to detect apoptosis. Granulocyte differentiation was determined (Table S1) from the Wright Giemsa staining and the NTB reduction assay.

The library of styryl quinazolinones was structurally differentiated at three parts of the scaffold, namely the phenyl rings at R2 and R3, and the quinazolinone ring R1 (Scheme 1B). Granulocyte differentiation was affected by the substitution of the heteroaryl ring R1. With the exception of the 6-fluoro substitution, other groups such as the iodo derivative, **32**, dimethoxy derivatives **20**, **42**, **50**, **54**, **55**, **58**, and dichloro derivative **51** were generally not favored. There was a general preference for hydrophobic groups at R2, such as **1** (2-methoxy Ph), **25** (3-methoxy Ph), **62** (3,4,5-trimethoxy Ph), **66** (4-carboxy methyl Ph), **68**, and **79** (4-fluoro Ph). On the other hand, polar OH groups were preferred at R3. Replacing phenyl R3 with a furan ring, preferably substituted with 2-nitro, improved activity. The stereochemistry of the ethenyl linker, connecting ring R3 to the quinazolinone scaffold, was critical and should be trans.

**Figure 2:**
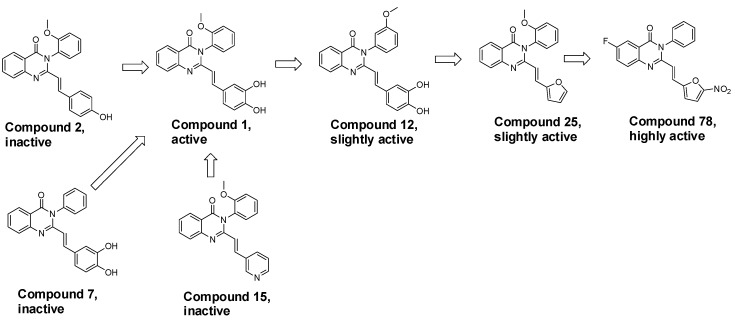
Schematic showing the potent compound **78** and the structure-based variation in differentiation of HL-60.

These general SAR trends are illustrated in Figure 2 with specific examples. Compound **2** with ortho-MeO at ring R2 and mono-OH at ring R3 was inactive, but activity was restored when ring 3 was substituted with two OH groups in **1**. Interestingly, retaining this motif and moving the methoxy on ring R2 from ortho to meta, decreased activity (**12**). Two other inactive analogs related to **1** were **7** and **15**. In **7**, ring R2 was not substituted, and in **15**, ring R3 was replaced with pyridyl. These marked changes in activity suggests, firstly, that the substitution on both rings R2 and R3 is important, and secondly, that there is a critical dependency between the two substitution patterns that remains to be determined. Activities of the compounds **1**, **12**, and **25**, provide further confirmation of this inference. Keeping the seemingly favored ortho OMe on ring R2 of **25**, while replacing the phenyl ring R3 with furan, led to some loss in activity. Inserting a nitro substituent to the furan ring R3 of **25** resulted in potent analogues **66**, **67**, **78**, and **79**.

**Figure 3:**
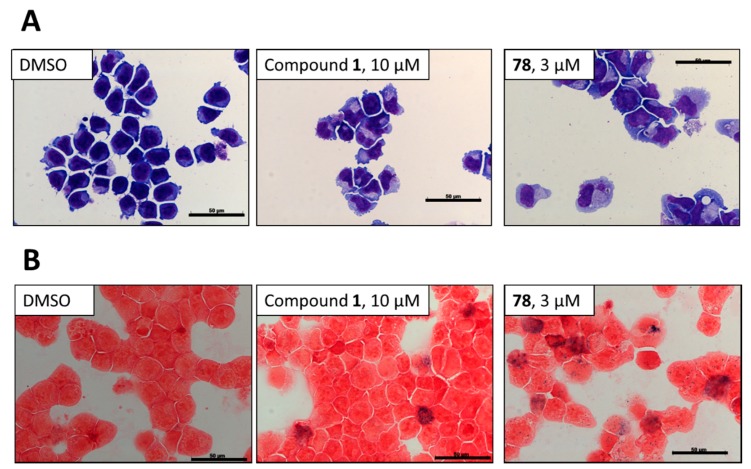
Styryl quinazolinone derivatives induce granulocytic differentiation and are determined by (**A**) Wright-Giemsa staining for morphology, and (**B**) NBT assay for function. HL-60 cells were treated with 0.1% DMSO, 10 μM compound **1**, or 3 μM compound **78** for seven days. Bars = 50 μm.

Compound **78**, with 6-fluoro substitution at the quinazolinone ring R1, and leaving ring R2 unsubstituted (seen in **7** to be abolished activity), was found to be the most active analogue identified from our screening efforts. Styryl quinazolinone **78** at 3 µM induced granulocytic differentiation in HL-60 cells, upon treatment for seven days, as characterized by a decreased nucleus-cytoplasm ratio, the presence of granules, and nuclear segmentation (Figure 3A). Similar granulocytic differentiation was observed with 10 µM of **1**, whereas the negative control Dimethyl Sulfoxide (DMSO) showed no characteristic differentiation. In the NBT reduction assay to detect the production of superoxide anion, 55% were positive in **78** treated cells, compared to 0–5% in DMSO treated control cells (Figure 3B). However, other potent analogues, **66**, **67**, and **79**, lost activities at 3 µM (0–5%). Even with 6-fluoro quinazolinone substitution, the derivatives **67**, **70**, **72**, **75**, **79**, and **80** were found to be less active towards inducing differentiation compared to **78**.

**Figure 4:**
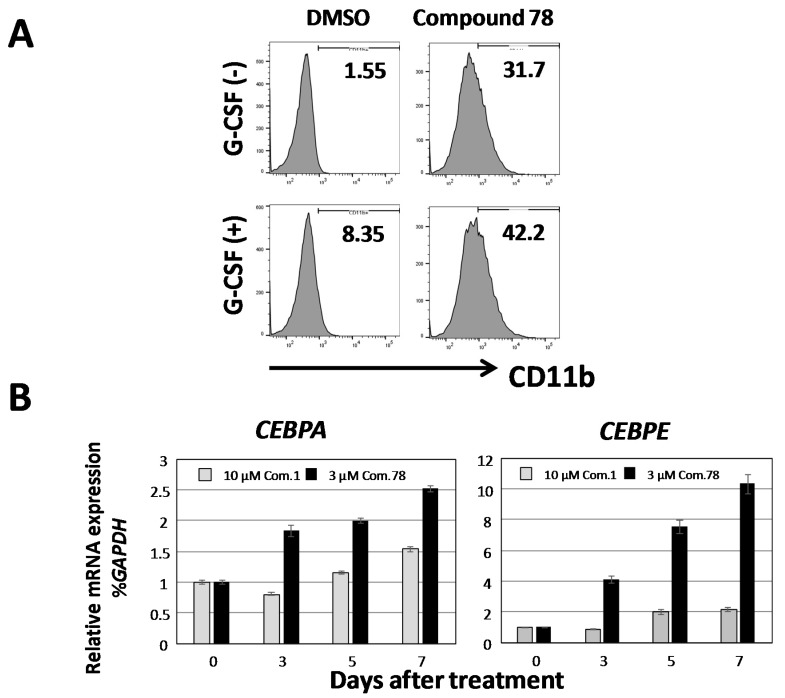
Compound **78** induces the expression of the neutrophil differentiation surface marker CD11b and upregulation of CEBPA and its downstream targets: (**A**) CD11b positive cells were evaluated by flow cytometry analysis; and (**B**) expression of *CEBPA* and *CEBPE* was determined by quantitative real time PCR analysis.

Additionally, compound **78** increased the expression of the neutrophil differentiation surface marker CD11b (Figure 4A). Similar results were obtained using MOLM-14 cells, which were established from a patient with acute monocytic leukemia (M5a) (Supplementary Figure S1). We have reported that **1** and granulocyte-colony stimulating factor (G-CSF) synergistically enhance granulocytic differentiation in HL-60 cells [21]. As expected, G-CSF enhanced the expression of CD11b, which was induced by compound **78** (31.7% vs. 42.2%, Figure 3A). These results suggest that **78** can upregulate G-CSF receptors, and render leukemic cells more sensitive to G-CSF stimulation.

Further, we examined the gene expression levels of the C/EBPα gene (*CEBPA*) and the C/EBPε gene (*CEBPE*) [21]—one of the downstream target genes of C/EBPα. When HL-60 cells were treated with **78**, mRNA expression levels of *CEBPA* and *CEBPE* were increased in a time-dependent manner (Figure 4B). We observed that **78** could induce the increase of *C/EBPα* (2.5-fold) and *C/EBPε* (10.3-fold) by day seven. These results further demonstrate that **78** induces granulocytic differentiation in leukemia cell lines, which is mediated through upregulation of C/EBPα.

## 3. Conclusions

In this study we have identified the derivative **78** as a potent inducer of granulocytic differentiation in HL-60 cells—apart from **1**. The improvement in potency may be mainly due to the introduction of the 5-nitro furan group at ring R3. This modification not only improves the granulocytic differentiation ability of the styryl quinazolinones, but also replaces the Pan Assay Interference compounds (PAINS) susceptible moiety 3,4-dihydroxy phenyl group in compound **1**. The other factor influencing the case of **78** can be the fluoro substitution of the quinazolinone scaffold. Overall, we have identified a new and improved inducer for myeloid differentiation in HL-60 cells by suitably modifying the 3,4-dihydroxy phenyl part of **1**, with the 5-nitrofuran group, which can be further developed as a potential drug targeting inducer of C/EBPα expression for leukemia treatment. To the best of our knowledge, this is the first report describing the structure activity relations of styryl quinazolinones relating to the induction of C/EBPα expression, and during the investigation we found a lead, like molecule **78**, with more promising activity (at 3 μM concentration) than our earlier lead, compound **1**. We strongly believe that repurposing styryl quinazolnines, for inducing C/EBPα expression in AML, would rejuvenate further research on these types of small molecules.

## 4. Materials and Methods

**Reagents:** All-trans retinoic acid (ATRA) was purchased from Sigma-Aldrich (R2625, St Louis, MO, USA). Compounds **1** to **60** were purchased from the ICCB-Longwood Screening Facility at Harvard Medical School. Compounds **61** to **80** were obtained from Indian Institute of Chemical Technology and the synthesis of these compounds were reported earlier [20]. 2-[*E*-2-(3,4-dihydroxyphenyl), vinyl]-3-(2-methoxyphenyl)-4(3*H*)-quinazolinone, compound 1, and 6-fluoro-2-[(*E*)-2-(5-nitrofuran-2-yl)ethenyl]-3-phenylquinazolin-4(3*H*)-one (78) were resynthesized (Scheme 1) at the National University of Singapore. Stock solutions for the compounds were solved in DMSO and stored at −20 °C. These compounds were diluted in a fresh complete medium before the experiment, and the final concentration of DMSO was 0.1%. Human G-SCF was purchased from Miltenyl Biotec (130-096-345, Bergisch Gladbach, Germany). Human G-CSF was prepared in 0.1% bovine serum albumin/PBS, and stored at −20 °C.

**Cells:** HL-60 (CCL-240) cells were purchased from American Type Culture Collection (ATCC, Manassas, VA, USA). MOLM-14 cells were kindly provided by Dr. Gary Gililand. These cell lines were maintained in RPMI 1640 with 10% fetal bovine serum at 37 °C with 5% CO_2_.

**Morphological Analysis and Nitro Blue Tetrazolium Reduction Assay:** HL-60 cells were treated with 0.1% DMSO, 10 µM of 1, and 3 µM of **78** for 7 days and stained with Wright-Giemsa. Maximum cell concentrations were adjusted lower than 100,000 /mL. These treated HL-60 cells were incubated with 1.0 mg/mL nitroblue tetrazolium/PBS, and 0.36 µM Phorbol-12-myristate-13-acetate (PMA) at 37 °C for 30–40 min and stained with safranin O. Morphological differentiation and apoptosis were evaluated as − (0–5%), + (5–30%), and ++ (more than 30%), as previously described [21].

**Flow Cytometry:** HL-60 cells were treated with 0.1% DMSO, 10 µM of 1, and 3 µM of **78** with or without 60 ng/mL of human G-CSF for 7 days and were stained with APC anti-mouse/human CD11b antibody from Biolegend (101211, San Diego, CA), and was analyzed by LSRII flow (BD Biosciences, Franklin Lakes, NJ, USA). We used FlowJo (FLOWJO, LLC, Ashland, OR, USA) to analyze the data.

**Quantitative Real-Time PCR (QT-PCR):** Total RNA was isolated from HL-60 cells using RNeasy Mini Kit (Qiagen, Valencia, CA, USA). Complementary DNA was synthesized using SuperScript II (Thermo Fisher Scientific, Waltham, MA, USA). The mRNA levels of genes were measured by QT-PCR using the Rotor Gene 6000 sequence detection system (Qiagen, Valencia, CA, USA) with iTaq Universal SYBR Green Supermix (BIO-RAD, Hercules, CA, USA). The expression levels of the glyceraldehyde 3-phosohate dehydrogenase (GAPDH) were used for normalization. Reactions were performed in technical triplicates. The primers for qPCR, human C/EBP*α* sense 5′-TGTGCCTTGGAAATGCAAAC-3′, antisense 5′-CGGGAAGGAGGCAGGAAA-3′, human C/EBP*ε* sense 5′-GCGTTCTCAAGGCCCCTT-3′, antisense 5′-GGGAGGGCGCCTTCAG-3′, human GAPDH sense 5′-CCACATCGCTCAGACACCAT-3′, and antisense 5′-CCAGGCGCCCAATACG-3′.

**Statistical Analysis:** Differences between the experimental groups were tested with Student’s *t*-test. *p*-values of less than 0.05 were considered statistically significant.

## Supplementary Materials

The following are available online, Table S1: Styryl quinazolinone derivatives evaluated in this study. Figure S1: Compound 78 induces morphological changes in MOLM-14 cells.
