# Supplementary material for: Styryl Quinazolinones as Potential Inducers of Myeloid Differentiation via Upregulation of C/EBPα
Source: Molecules. 2018 Aug 3;23(8):1938. doi: 10.3390/molecules23081938 (PMC6222906; doi:10.3390/molecules23081938)
Supplement: Supplementary file 1 [file molecules-23-01938-s001.zip › molecules-326132-supplementary-final/Sup Figure S1.pdf]

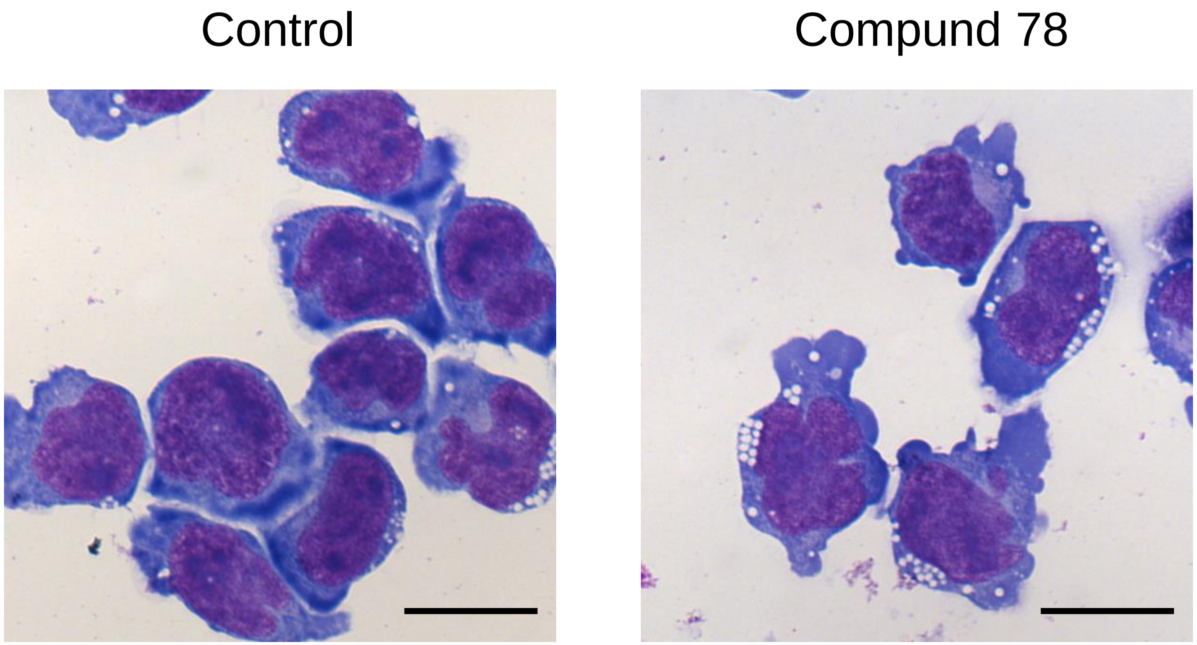

Supplementary Figure S1. Compound78 induces morphological changes in MOLM-14 cells. Cells were treated with either 0.1% DMSO or 3 µM compound 78 for 7 days. Cells were cytospinned and subjected to wright-giemsa staining. Bars = 10 µm.
