# Supplementary material for: Styryl Quinazolinones as Potential Inducers of Myeloid Differentiation via Upregulation of C/EBPα
Source: Molecules. 2018 Aug 3;23(8):1938. doi: 10.3390/molecules23081938 (PMC6222906; doi:10.3390/molecules23081938)
Supplement: Supplementary file 1 [file molecules-23-01938-s001.zip › molecules-326132-supplementary-final/Sup Table S1.pdf]

Table S1. Styryl Quinazolinone derivatives (**1** to **80**).

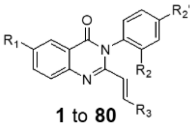

1 to **80**

Entry	Structure	Name of compound	Differentiation at 10 μM	apoptosis at 10 μM
**1**	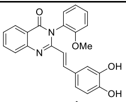	2- [(*E*) - 2- (3,4- dihydroxyphenyl) vinyl]- 3- (2- methoxyphenyl) quinazolin- 4(3*H*) - one	++	++
**2**	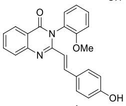	2- [(*E*) - 2- (4- hydroxyphenyl) vinyl]- 3- (2- methoxyphenyl) quinazolin- 4(3*H*) - one	–	–
**3**	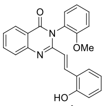	2- [(*E*) - 2- (2- hydroxyphenyl) vinyl]- 3- (2- methoxyphenyl) quinazolin- 4(3*H*) - one	–	–
**4**	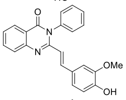	2- [(*E*) - 2- (4- hydroxy- 3- methoxyphenyl) vinyl]- 3- phenylquinazolin- 4(3*H*) - one	–	–
**5**	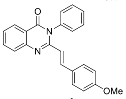	2- [(*E*) - 2- (4- methoxyphenyl) vinyl]- 3- phenylquinazolin- 4(3*H*) - one	–	–
**6**	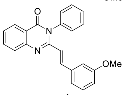	2- [(*E*) - 2- (3- methoxyphenyl) vinyl]- 3- phenylquinazolin- 4(3*H*) - one	–	–
**7**	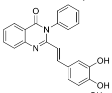	2- [(*E*) - 2- (3,4- dihydroxyphenyl) vinyl]- 3- phenylquinazolin- 4(3*H*) - one	–	–
**8**	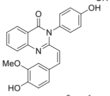	2- [(*Z*) - 2- (4- hydroxy- 3- methoxyphenyl) vinyl]- 3- (4- hydroxyphenyl) quinazolin- 4(3*H*) - one	+	+
**9**	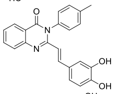	2- [(*E*) - 2- (3,4- dihydroxyphenyl) vinyl]- 3- (4- methylphenyl) quinazolin- 4(3*H*) - one	–	–
**10**	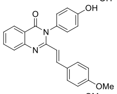	3- (4- hydroxyphenyl) - 2- [(*E*) - 2- (4- methoxyphenyl) vinyl]quinazolin- 4(3*H*) - one	–	–
**11**	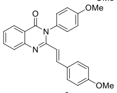	3- (4- methoxyphenyl) - 2- [(*E*) - 2- (4- methoxyphenyl) vinyl]quinazolin- 4(3*H*) - one	–	–
**12**	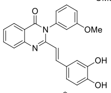	2- [(*E*) - 2- (3,4- dihydroxyphenyl) vinyl]- 3- (3- methoxyphenyl) quinazolin- 4(3*H*) - one	+	+
**13**	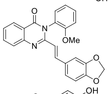	2- [(*E*) - 2- (1,3- benzodioxol- 5- yl) vinyl]- 3- (2- methoxyphenyl) quinazolin- 4(3*H*) - one	–	–
**14**	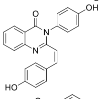	3- (4- hydroxyphenyl) - 2- [(*Z*) - 2- (4- hydroxyphenyl) vinyl]quinazolin- 4(3*H*) - one	–	–
**15**	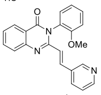	3- (2- methoxyphenyl) - 2- [(*E*) - 2- pyridin- 3- ylvinyl]quinazolin- 4(3*H*) - one	–	–
**16**	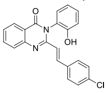	2- [(*E*) - 2- (4- chlorophenyl) vinyl]- 3- (2- hydroxyphenyl) quinazolin- 4(3*H*) - one	–	–

**17**	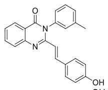	2- [(*E*) - 2- (4- hydroxyphenyl) vinyl]- 3- (3- methylphenyl) quinazolin- 4(3*H*) - one	−	−
**18**	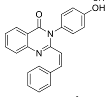	3- (4- hydroxyphenyl) - 2- [(*Z*) - 2- phenylvinyl]quinazolin- 4(3*H*) - one	−	−
**19**	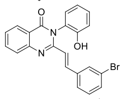	2- [(*E*) - 2- (3- bromophenyl) vinyl]- 3- (2- hydroxyphenyl) quinazolin- 4(3*H*) - one	−	−
**20**	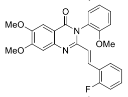	2- [(*E*) - 2- (2- fluorophenyl) vinyl]- 6,7- dimethoxy- 3- (2- methoxyphenyl) quinazolin- 4(3*H*) - one	−	−
**21**	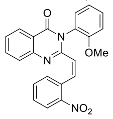	3- (2- methoxyphenyl) - 2- [(*Z*) - 2- (2- nitrophenyl) vinyl]quinazolin- 4(3*H*) - one	−	−
**22**	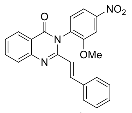	3- (2- methoxy- 4- nitrophenyl) - 2- [(*E*) - 2- phenylvinyl]quinazolin- 4(3*H*) - one	−	−
**23**	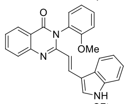	2- [(*E*) - 2- (1*H*- indol- 3- yl) vinyl]- 3- (2- methoxyphenyl) quinazolin- 4(3*H*) - one	−	−
**24**	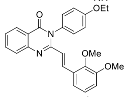	2- [(*E*) - 2- (2,3- dimethoxyphenyl) vinyl]- 3- (4- ethoxyphenyl) quinazolin- 4(3*H*) - one	−	−
**25**	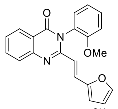	2- [(*E*) - 2- (2- furyl) vinyl]- 3- (2- methoxyphenyl) quinazolin- 4(3*H*) - one	+	+
**26**	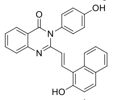	2- [(*E*) - 2- (2- hydroxy- 1- naphthyl) vinyl]- 3- (4- hydroxyphenyl) quinazolin- 4(3*H*) - one	−	−
**27**	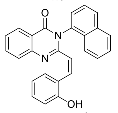	2- [(*Z*) - 2- (2- hydroxyphenyl) vinyl]- 3- (1- naphthyl) quinazolin- 4(3*H*) - one	−	−
**28**	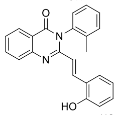	2- [(*E*) - 2- (2- hydroxyphenyl) vinyl]- 3- (2- methylphenyl) quinazolin- 4(3*H*) - one	−	+
**29**	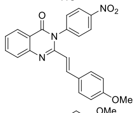	2- [(*E*) - 2- (4- methoxyphenyl) vinyl]- 3- (4- nitrophenyl) quinazolin- 4(3*H*) - one	−	−
**30**	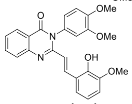	3- (3,4- dimethoxyphenyl) - 2- [(*E*) - 2- (2- hydroxy- 3- methoxyphenyl) vinyl]quinazolin- 4(3*H*) - one	−	−
**31**	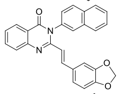	2- [(*E*) - 2- (1,3- benzodioxol- 5- yl) vinyl]- 3- (2- naphthyl) quinazolin- 4(3*H*) - one	−	−
**32**	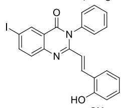	2- [(*E*) - 2- (2- hydroxyphenyl) vinyl]- 6- iodo- 3- phenylquinazolin- 4(3*H*) - one	−	−
**33**	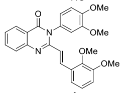	3- (3,4- dimethoxyphenyl) - 2- [(*E*) - 2- (2,3- dimethoxyphenyl) vinyl]quinazolin- 4(3*H*) - one	−	−
**34**	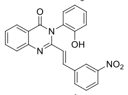	3- (2- hydroxyphenyl) - 2- [(*E*) - 2- (3- nitrophenyl) vinyl]quinazolin- 4(3*H*) - one	−	−
**35**	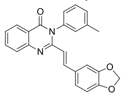	2- [(*E*) - 2- (1,3- benzodioxol- 5- yl) vinyl]- 3- (3- methylphenyl) quinazolin- 4(3*H*) - one	+	−

**36**	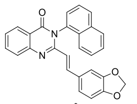	2- [(*E*) - 2- (1,3- benzodioxol- 5- yl) vinyl]- 3- (1- naphthyl) quinazolin- 4(3*H*) - one	−	−
**37**	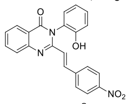	3- (2- hydroxyphenyl) - 2- [(*E*) - 2- (4- nitrophenyl) vinyl]quinazolin- 4(3*H*) - one	−	−
**38**	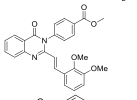	methyl 4- {2- [(*E*) - 2- (2,3- dimethoxyphenyl) vinyl]- 4- oxoquinazolin- 3(4*H*) - yl}benzoate	−	−
**39**	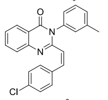	2- [(*Z*) - 2- (4- chlorophenyl) vinyl]- 3- (3- methylphenyl) quinazolin- 4(3*H*) - one	−	−
**40**	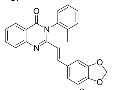	2- [(*E*) - 2- (1,3- benzodioxol- 5- yl) vinyl]- 3- (2- methylphenyl) quinazolin- 4(3*H*) - one	−	−
**41**	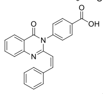	4- {4- oxo- 2- [(*Z*) - 2- phenylvinyl]quinazolin- 3(4*H*) - yl}benzoic acid	−	−
**42**	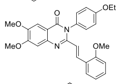	3- (4- ethoxyphenyl) - 6,7- dimethoxy- 2- [(*E*) - 2- (2- methoxyphenyl) vinyl]quinazolin- 4(3*H*) - one	−	−
**43**	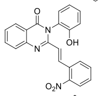	3- (2- hydroxyphenyl) - 2- [(*E*) - 2- (2- nitrophenyl) vinyl]quinazolin- 4(3*H*) - one	−	−
**44**	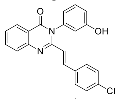	2- [(*E*) - 2- (4- chlorophenyl) vinyl]- 3- (3- hydroxyphenyl) quinazolin- 4(3*H*) - one	−	−
**45**	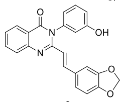	2- [(*E*) - 2- (1,3- benzodioxol- 5- yl) vinyl]- 3- (3- hydroxyphenyl) quinazolin- 4(3*H*) - one	−	−
**46**	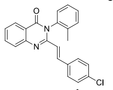	2- [(*E*) - 2- (4- chlorophenyl) vinyl]- 3- (2- methylphenyl) quinazolin- 4(3*H*) - one	−	−
**47**	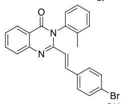	2- [(*E*) - 2- (4- bromophenyl) vinyl]- 3- (2- methylphenyl) quinazolin- 4(3*H*) - one	−	−
**48**	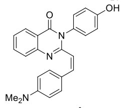	2- {(*Z*) - 2- [4- (dimethylamino) phenyl]vinyl}- 3- (4- hydroxyphenyl) quinazolin- 4(3*H*) - one	−	+
**49**	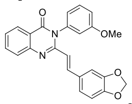	2- [(*E*) - 2- (1,3- benzodioxol- 5- yl) vinyl]- 3- (3- methoxyphenyl) quinazolin- 4(3*H*) - one	−	+
**50**	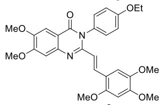	3- (4- ethoxyphenyl) - 6,7- dimethoxy- 2- [(*E*) - 2- (2,4,5- trimethoxyphenyl) vinyl]quinazolin- 4(3*H*) - one	−	−
**51**	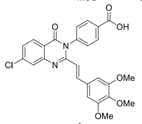	4- {7- chloro- 4- oxo- 2- [(*E*) - 2- (3,4,5- trimethoxyphenyl) vinyl]quinazolin- 3(4*H*) - yl}benzoic acid	−	−
**52**	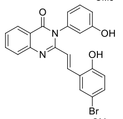	2- [(*E*) - 2- (5- bromo- 2- hydroxyphenyl) vinyl]- 3- (3- hydroxyphenyl) quinazolin- 4(3*H*) - one	−	−
**53**	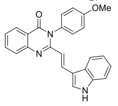	2- [(*E*) - 2- (1*H*- indol- 3- yl) vinyl]- 3- (4- methoxyphenyl) quinazolin- 4(3*H*) - one	−	−
**54**	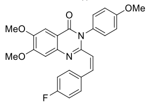	2- [(*Z*) - 2- (4- fluorophenyl) vinyl]- 6,7- dimethoxy- 3- (4- methoxyphenyl) quinazolin- 4(3*H*) - one	−	−

**55**	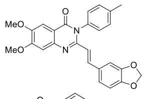	2- [(*E*) - 2- (1,3- benzodioxol- 5- yl) vinyl]- 6,7- dimethoxy- 3- (4- methylphenyl) quinazolin- 4(3*H*) - one	−	−
**56**	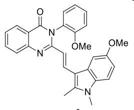	2- [(*E*) - 2- (5- methoxy- 1,2- dimethyl- 1H- indol- 3- yl) vinyl]- 3- (2- methoxyphenyl) quinazolin- 4(3*H*) - one	−	−
**57**	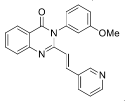	3- (3- methoxyphenyl) - 2- [(*E*) - 2- pyridin- 3- ylvinyl]quinazolin- 4(3*H*) - one	−	−
**58**	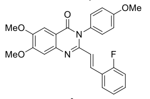	2- [(*E*) - 2- (2- fluorophenyl) vinyl]- 6,7- dimethoxy- 3- (4- methoxyphenyl) quinazolin- 4(3*H*) - one	−	+
**59**	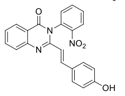	2- [(*E*) - 2- (4- hydroxyphenyl) vinyl]- 3- (2- nitrophenyl) quinazolin- 4(3*H*) - one	−	−
**60**	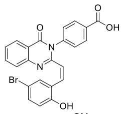	4- {2- [(*Z*) - 2- (5- bromo- 2- hydroxyphenyl) vinyl]- 4- oxoquinazolin- 3(4*H*) - yl}benzoic acid	−	−
**61**	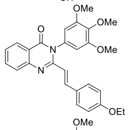	2- [(*E*) - 2- (4- ethoxyphenyl) vinyl]- 3- (3,4,5- methoxyphenyl) quinazolin- 4(3*H*) - one	-	-
**62**	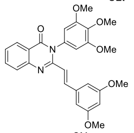	2- [(*E*) - 2- (3,5-dimethoxyphenyl) vinyl]- 3- (3,4,5- methoxyphenyl) quinazolin- 4(3*H*) - one	+	+
**63**	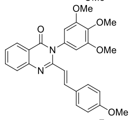	2- [(*E*) - 2- (4-methoxyphenyl) vinyl]- 3- (3,4,5- methoxyphenyl) quinazolin- 4(3*H*) - one	-	-
**64**	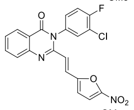	2- [(*E*) - 2- (5-nitro furan-2-yl) vinyl]- 3- (4-Fluoro-3-Chlorophenyl) quinazolin- 4(3*H*) - one	-	+
**65**	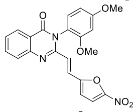	2- [(*E*) - 2- (5-nitro furan-2-yl) vinyl]- 3- (2,4-dimethoxy phenyl) quinazolin- 4(3*H*) - one	-	-
**66**	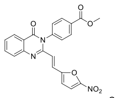	(*E*)-methyl 4-(2-(2-(5-nitrofuran-2-yl)vinyl)-4-oxoquinazolin-3(4*H*)-yl)benzoate	++	+
**67**	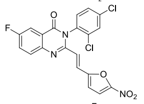	2- [(*E*) - 2- (5-nitro furan-2-yl) vinyl]- 6-fluoro-3- (2,4-dichloro phenyl) quinazolin- 4(3*H*) - one	-	+
**68**	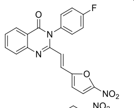	2- [(*E*) - 2- (5-nitro furan-2-yl) vinyl]-3- (4-Fluoro phenyl) quinazolin- 4(3*H*) - one	++	+
**69**	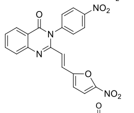	2- [(*E*) - 2- (5-nitro furan-2-yl) vinyl]-3- (4-nitro phenyl) quinazolin- 4(3*H*) - one	-	+
**70**	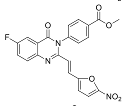	(*E*)-methyl 4-(6-fluoro-2-(2-(5-nitrofuran-2-yl)vinyl)-4-oxoquinazolin-3(4*H*)-yl)benzoate	+	+
**71**	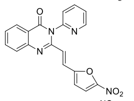	2- [(*E*) - 2- (5-nitro furan-2-yl) vinyl]-3- (pyridin-2-yl) quinazolin- 4(3*H*) - one	-	-
**72**	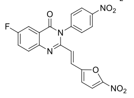	2- [(*E*) - 2- (5-nitro furan-2-yl) vinyl]-6-fluoro-3- (4-nitro phenyl) quinazolin- 4(3*H*) - one	-	+

**73**	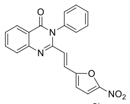	2- [(*E*) - 2- (5-nitro furan-2-yl) vinyl]-3- (phenyl) quinazolin- 4(3*H*) - one	-	-
**74**	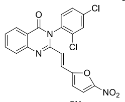	2- [(*E*) - 2- (5-nitro furan-2-yl) vinyl]-3- (2,4-dichloro phenyl) quinazolin- 4(3*H*) - one	-	-
**75**	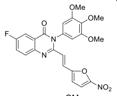	2- [(*E*) - 2- (5-nitro furan-2-yl) vinyl]-6-fluoro-3- (3,4,5-trimethoxy phenyl) quinazolin- 4(3*H*) - one	-	+
**76**	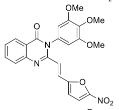	2- [(*E*) - 2- (5-nitro furan-2-yl) vinyl]-3- (3,4,5-trimethoxy phenyl) quinazolin- 4(3*H*) - one	+	-
**77**	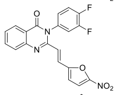	2- [(*E*) - 2- (5-nitro furan-2-yl) vinyl]-3- (3,4-difluoro phenyl) quinazolin- 4(3*H*) - one	-	-
**78**	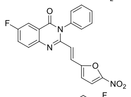	2- [(*E*) - 2- (5-nitro furan-2-yl) vinyl]-6-fluoro-3- (phenyl) quinazolin- 4(3*H*) - one	++	+
**79**	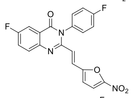	2- [(*E*) - 2- (5-nitro furan-2-yl) vinyl]-6-fluoro-3- (4-fluoro phenyl) quinazolin- 4(3*H*) - one	++	+
**80**	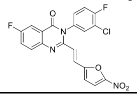	2- [(*E*) - 2- (5-nitro furan-2-yl) vinyl]-6-fluoro-3- (4-fluoro-3-chloro phenyl) quinazolin- 4(3*H*) - one	-	+
